# Supplementary material for: Incidence and temporal changes in lumbar degeneration and low back pain in child and adolescent weightlifters: A prospective 5-year cohort study
Source: PLoS One. 2022 Jun 29;17(6):e0270046. doi: 10.1371/journal.pone.0270046 (PMC9242517; doi:10.1371/journal.pone.0270046)
Supplement: Attachment — Submitted filename: Response_to_Reviewers.docx [file pone.0270046.s003.docx]

PONE-D-21-34347R1

Incidence and temporal changes in lumbar degeneration and low back pain in child and adolescent weightlifters: A prospective 5-year cohort study

PLOS ONE

Dear Dr. Nakase,

Thank you for submitting your manuscript to PLOS ONE. After careful consideration, we feel that it has merit but does not fully meet PLOS ONE’s publication criteria as it currently stands. Therefore, we invite you to submit a revised version of the manuscript that addresses the points raised during the review process.
Please submit your revised manuscript by Jun 04 2022 11:59PM. If you will need more time than this to complete your revisions, please reply to this message or contact the journal office at [plosone@plos.org](mailto:plosone@plos.org). When you're ready to submit your revision, log on to https://www.editorialmanager.com/pone/ and select the 'Submissions Needing Revision' folder to locate your manuscript file.

Please include the following items when submitting your revised manuscript:

- A rebuttal letter that responds to each point raised by the academic editor and reviewer(s). You should upload this letter as a separate file labeled 'Response to Reviewers'.
- A marked-up copy of your manuscript that highlights changes made to the original version. You should upload this as a separate file labeled 'Revised Manuscript with Track Changes'.
- An unmarked version of your revised paper without tracked changes. You should upload this as a separate file labeled 'Manuscript'.

If you would like to make changes to your financial disclosure, please include your updated statement in your cover letter. Guidelines for resubmitting your figure files are available below the reviewer comments at the end of this letter.

If applicable, we recommend that you deposit your laboratory protocols in protocols.io to enhance the reproducibility of your results. Protocols.io assigns your protocol its own identifier (DOI) so that it can be cited independently in the future. For instructions see: <https://journals.plos.org/plosone/s/submission-guidelines#loc-laboratory-protocols>. Additionally, PLOS ONE offers an option for publishing peer-reviewed Lab Protocol articles, which describe protocols hosted on protocols.io. Read more information on sharing protocols at <https://plos.org/protocols?utm_medium=editorial-email&utm_source=authorletters&utm_campaign=protocols>.

We look forward to receiving your revised manuscript.

Kind regards,

Svenja Illien-Jünger, Ph.D.
Academic Editor
PLOS ONE

Journal Requirements:

Please review your reference list to ensure that it is complete and correct. If you have cited papers that have been retracted, please include the rationale for doing so in the manuscript text, or remove these references and replace them with relevant current references. Any changes to the reference list should be mentioned in the rebuttal letter that accompanies your revised manuscript. If you need to cite a retracted article, indicate the article’s retracted status in the References list and also include a citation and full reference for the retraction notice.

[Note: HTML markup is below. Please do not edit.]

Reviewers' comments:

Reviewer's Responses to Questions

**Comments to the Author**

1. If the authors have adequately addressed your comments raised in a previous round of review and you feel that this manuscript is now acceptable for publication, you may indicate that here to bypass the “Comments to the Author” section, enter your conflict of interest statement in the “Confidential to Editor” section, and submit your "Accept" recommendation.

Reviewer #1: All comments have been addressed

Reviewer #2: (No Response)

2. Is the manuscript technically sound, and do the data support the conclusions?

The manuscript must describe a technically sound piece of scientific research with data that supports the conclusions. Experiments must have been conducted rigorously, with appropriate controls, replication, and sample sizes. The conclusions must be drawn appropriately based on the data presented.

Reviewer #1: Yes

Reviewer #2: Partly

3. Has the statistical analysis been performed appropriately and rigorously?

Reviewer #1: Yes

Reviewer #2: No

4. Have the authors made all data underlying the findings in their manuscript fully available?

The [PLOS Data policy](http://www.plosone.org/static/policies.action#sharing) requires authors to make all data underlying the findings described in their manuscript fully available without restriction, with rare exception (please refer to the Data Availability Statement in the manuscript PDF file). The data should be provided as part of the manuscript or its supporting information, or deposited to a public repository. For example, in addition to summary statistics, the data points behind means, medians and variance measures should be available. If there are restrictions on publicly sharing data—e.g. participant privacy or use of data from a third party—those must be specified.

Reviewer #1: Yes

Reviewer #2: Yes

5. Is the manuscript presented in an intelligible fashion and written in standard English?

PLOS ONE does not copyedit accepted manuscripts, so the language in submitted articles must be clear, correct, and unambiguous. Any typographical or grammatical errors should be corrected at revision, so please note any specific errors here.

Reviewer #1: Yes

Reviewer #2: Yes

6. Review Comments to the Author

Please use the space provided to explain your answers to the questions above. You may also include additional comments for the author, including concerns about dual publication, research ethics, or publication ethics. (Please upload your review as an attachment if it exceeds 20,000 characters)

Reviewer #1: The authors adequately addressed my previous comments. While the studies does possess some flaws, these were appropriately addressed in the limitations section. I recommend acceptance as the positive aspects of the study outweigh potential flaws.

→ Thank you very much for reviewing our manuscript.

Reviewer #2: The authors have shown a good attempt to address all previous comments and concerns with the manuscript. However, additional concerns are still present, as outlined below.

Line 63- Consider changing to ‘weightlifting training’ rather than S&C.

→ Thank you for pointing this out. We have corrected the text (Line 61).

Line 97- Remove ‘no’ prior to history.

→ Thank you for pointing this out. We have corrected the text (Line 92).

Line 134- Edits suggest that statistical analysis was conducted (in SPSS), however it is mentioned in the limitations that this was not possible (line 280). I would argue the lack of statistical analysis is still downplayed, despite being a major limitation of the study.
→ As you pointed out, we believe that the most significant limitation of this study is the lack of statistical analyses. We have removed the sentence about using SPSS (Line 126).

Line 142- Consider changing to ‘One Repetition Maximum (1RM)’ to ensure clarity. The prescription of these loads seems rather arbitrary. Are you able to provide a reference to support this prescription?

→ We have made the required revisions in accordance with your suggestion (Line 133-134). As mentioned in the Discussion section, the training load on the participants is likely to be lower than that recommended by the guidelines, while the training frequency is likely to be higher. We were unable to find any literature to support such a training prescription and, as you note, we must acknowledge that it was arbitrary. We have addressed this issue in the revised Limitations section (Line 284-289).


Line 306- The wording here is a little unclear. Consider referring to the fact that chronological age was used to assume maturity status. It would be useful to reference research supporting the limitations of this approach. Also, additional important information could be noted with regards to the training. Was technique assured throughout and were the sessions supervised by a qualified personal?

→ We have revised the text in accordance with your comments. We have also added information on the training prescription and supervision, based on the aforementioned information (Line 280-284).

7. PLOS authors have the option to publish the peer review history of their article ([what does this mean?](https://journals.plos.org/plosone/s/editorial-and-peer-review-process#loc-peer-review-history)). If published, this will include your full peer review and any attached files.


If you choose “no”, your identity will remain anonymous but your review may still be made public.


**Do you want your identity to be public for this peer review?** For information about this choice, including consent withdrawal, please see our [Privacy Policy](https://www.plos.org/privacy-policy).

Reviewer #1: No

Reviewer #2: No

[NOTE: If reviewer comments were submitted as an attachment file, they will be attached to this email and accessible via the submission site. Please log into your account, locate the manuscript record, and check for the action link "View Attachments". If this link does not appear, there are no attachment files.]

While revising your submission, please upload your figure files to the Preflight Analysis and Conversion Engine (PACE) digital diagnostic tool, <https://pacev2.apexcovantage.com/>. PACE helps ensure that figures meet PLOS requirements. To use PACE, you must first register as a user. Registration is free. Then, login and navigate to the UPLOAD tab, where you will find detailed instructions on how to use the tool. If you encounter any issues or have any questions when using PACE, please email PLOS at [figures@plos.org](mailto:figures@plos.org). Please note that Supporting Information files do not need this step.
